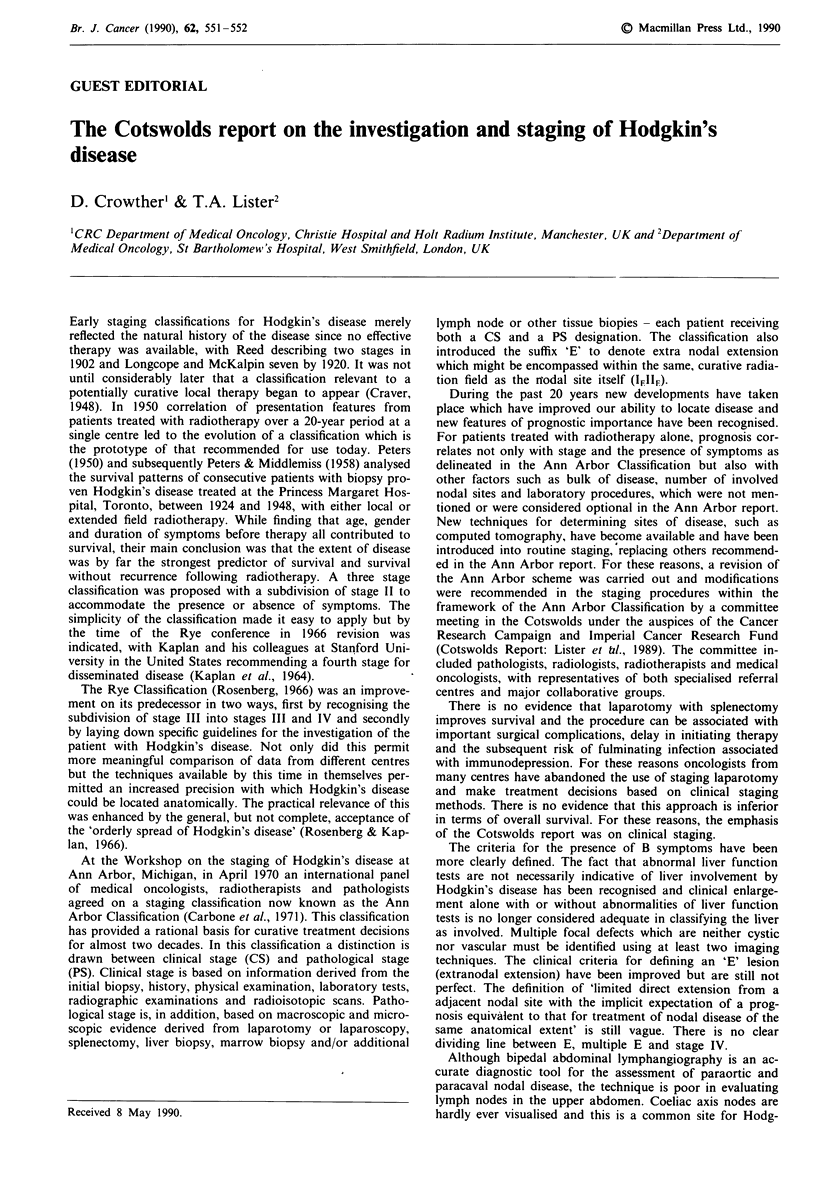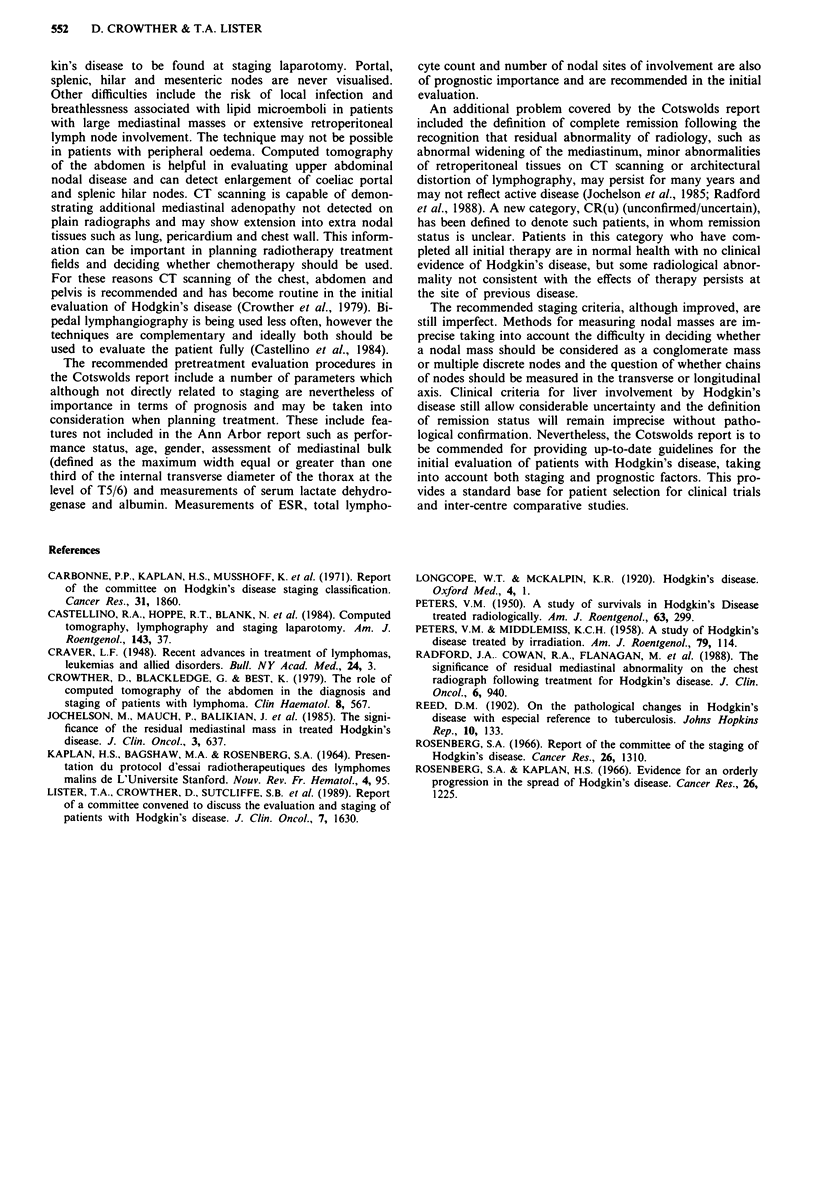# The Cotswolds report on the investigation and staging of Hodgkin's disease.

**DOI:** 10.1038/bjc.1990.328

**Published:** 1990-10

**Authors:** D. Crowther, T. A. Lister


 © Macmillan Press Ltd., 1990

GUEST EDITORIAL

### The Cotswolds report on the investigation and staging of Hodgkin's disease

D. Crowther[1] & T.A. Lister[2]

[1]CRC Department of Medical Oncology, Christie Hospital and Holt Radium Institute, Manchester, UK and [2]Department of Medical Oncology, St Bartholomew's Hospital, West Smithfield, London, UK

Early staging classifications for Hodgkin's disease merely reflected the natural history of the disease since no effective therapy was available, with Reed describing two stages in 1902 and Longcope and McKalpin seven by 1920. It was not until considerably later that a classification relevant to a potentially curative local therapy began to appear (Craver, 1948). In 1950 correlation of presentation features from patients treated with radiotherapy over a 20-year period at a single centre led to the evolution of a classification which is the prototype of that recommended for use today. Peters (1950) and subsequently Peters & Middlemiss (1958) analysed the survival patterns of consecutive patients with biopsy proven Hodgkin's disease treated at the Princess Margaret Hospital, Toronto, between 1924 and 1948, with either local or extended field radiotherapy. While finding that age, gender and duration of symptoms before therapy all contributed to survival, their main conclusion was that the extent of disease was by far the strongest predictor of survival and survival without recurrence following radiotherapy. A three stage classification was proposed with a subdivision of stage II to accommodate the presence or absence of symptoms. The simplicity of the classification made it easy to apply but by the time of the Rye conference in 1966 revision was indicated, with Kaplan and his colleagues at Stanford University in the United States recommending a fourth stage for disseminated disease (Kaplan *et al.*, 1964).

The Rye Classification (Rosenberg, 1966) was an improvement on its predecessor in two ways, first by recognising the subdivision of stage III into stages III and IV and secondly by laying down specific guidelines for the investigation of the patient with Hodgkin's disease. Not only did this permit more meaningful comparison of data from different centres but the techniques available by this time in themselves permitted an increased precision with which Hodgkin's disease could be located anatomically. The practical relevance of this was enhanced by the general, but not complete, acceptance of the 'orderly spread of Hodgkin's disease' (Rosenberg & Kaplan, 1966).

At the Workshop on the staging of Hodgkin's disease at Ann Arbor, Michigan, in April 1970 an international panel of medical oncologists, radiotherapists and pathologists agreed on a staging classification now known as the Ann Arbor Classification (Carbone *et al.*, 1971). This classification has provided a rational basis for curative treatment decisions for almost two decades. In this classification a distinction is drawn between clinical stage (CS) and pathological stage (PS). Clinical stage is based on information derived from the initial biopsy, history, physical examination, laboratory tests, radiographic examinations and radioisotopic scans. Pathological stage is, in addition, based on macroscopic and microscopic evidence derived from laparotomy or laparoscopy, splenectomy, liver biopsy, marrow biopsy and/or additional lymph node or other tissue biopies – each patient receiving both a CS and a PS designation. The classification also introduced the suffix 'E' to denote extra nodal extension which might be encompassed within the same, curative radiation field as the nodal site itself ($I_E II_E$).

During the past 20 years new developments have taken place which have improved our ability to locate disease and new features of prognostic importance have been recognised. For patients treated with radiotherapy alone, prognosis correlates not only with stage and the presence of symptoms as delineated in the Ann Arbor Classification but also with other factors such as bulk of disease, number of involved nodal sites and laboratory procedures, which were not mentioned or were considered optional in the Ann Arbor report. New techniques for determining sites of disease, such as computed tomography, have become available and have been introduced into routine staging, replacing others recommended in the Ann Arbor report. For these reasons, a revision of the Ann Arbor scheme was carried out and modifications were recommended in the staging procedures within the framework of the Ann Arbor Classification by a committee meeting in the Cotswolds under the auspices of the Cancer Research Campaign and Imperial Cancer Research Fund (Cotswolds Report: Lister *et al.*, 1989). The committee included pathologists, radiologists, radiotherapists and medical oncologists, with representatives of both specialised referral centres and major collaborative groups.

There is no evidence that laparotomy with splenectomy improves survival and the procedure can be associated with important surgical complications, delay in initiating therapy and the subsequent risk of fulminating infection associated with immunodepression. For these reasons oncologists from many centres have abandoned the use of staging laparotomy and make treatment decisions based on clinical staging methods. There is no evidence that this approach is inferior in terms of overall survival. For these reasons, the emphasis of the Cotswolds report was on clinical staging.

The criteria for the presence of B symptoms have been more clearly defined. The fact that abnormal liver function tests are not necessarily indicative of liver involvement by Hodgkin's disease has been recognised and clinical enlargement alone with or without abnormalities of liver function tests is no longer considered adequate in classifying the liver as involved. Multiple focal defects which are neither cystic nor vascular must be identified using at least two imaging techniques. The clinical criteria for defining an 'E' lesion (extranodal extension) have been improved but are still not perfect. The definition of 'limited direct extension from a adjacent nodal site with the implicit expectation of a prognosis equivalent to that for treatment of nodal disease of the same anatomical extent' is still vague. There is no clear dividing line between E, multiple E and stage IV.

Although bipedal abdominal lymphangiography is an accurate diagnostic tool for the assessment of paraortic and paracaval nodal disease, the technique is poor in evaluating lymph nodes in the upper abdomen. Coeliac axis nodes are hardly ever visualised and this is a common site for Hodg-

Received 8 May 1990.

kin's disease to be found at staging laparotomy. Portal, splenic, hilar and mesenteric nodes are never visualised. Other difficulties include the risk of local infection and breathlessness associated with lipid microemboli in patients with large mediastinal masses or extensive retroperitoneal lymph node involvement. The technique may not be possible in patients with peripheral oedema. Computed tomography of the abdomen is helpful in evaluating upper abdominal nodal disease and can detect enlargement of coeliac portal and splenic hilar nodes. CT scanning is capable of demonstrating additional mediastinal adenopathy not detected on plain radiographs and may show extension into extra nodal tissues such as lung, pericardium and chest wall. This information can be important in planning radiotherapy treatment fields and deciding whether chemotherapy should be used. For these reasons CT scanning of the chest, abdomen and pelvis is recommended and has become routine in the initial evaluation of Hodgkin's disease (Crowther *et al.*, 1979). Bipedal lymphangiography is being used less often, however the techniques are complementary and ideally both should be used to evaluate the patient fully (Castellino *et al.*, 1984).

The recommended pretreatment evaluation procedures in the Cotswolds report include a number of parameters which although not directly related to staging are nevertheless of importance in terms of prognosis and may be taken into consideration when planning treatment. These include features not included in the Ann Arbor report such as performance status, age, gender, assessment of mediastinal bulk (defined as the maximum width equal or greater than one third of the internal transverse diameter of the thorax at the level of T5/6) and measurements of serum lactate dehydrogenase and albumin. Measurements of ESR, total lymphocyte count and number of nodal sites of involvement are also of prognostic importance and are recommended in the initial evaluation.

An additional problem covered by the Cotswolds report included the definition of complete remission following the recognition that residual abnormality of radiology, such as abnormal widening of the mediastinum, minor abnormalities of retroperitoneal tissues on CT scanning or architectural distortion of lymphography, may persist for many years and may not reflect active disease (Jochelson *et al.*, 1985; Radford *et al.*, 1988). A new category, CR(u) (unconfirmed/uncertain), has been defined to denote such patients, in whom remission status is unclear. Patients in this category who have completed all initial therapy are in normal health with no clinical evidence of Hodgkin's disease, but some radiological abnormality not consistent with the effects of therapy persists at the site of previous disease.

The recommended staging criteria, although improved, are still imperfect. Methods for measuring nodal masses are imprecise taking into account the difficulty in deciding whether a nodal mass should be considered as a conglomerate mass or multiple discrete nodes and the question of whether chains of nodes should be measured in the transverse or longitudinal axis. Clinical criteria for liver involvement by Hodgkin's disease still allow considerable uncertainty and the definition of remission status will remain imprecise without pathological confirmation. Nevertheless, the Cotswolds report is to be commended for providing up-to-date guidelines for the initial evaluation of patients with Hodgkin's disease, taking into account both staging and prognostic factors. This provides a standard base for patient selection for clinical trials and inter-centre comparative studies.

### References

CARBONNE, P.P., KAPLAN, H.S., MUSSHOFF, K. *et al.* (1971). Report of the committee on Hodgkin's disease staging classification. *Cancer Res.*, **31**, 1860.

CASTELLINO, R.A., HOPPE, R.T., BLANK, N. *et al.* (1984). Computed tomography, lymphography and staging laparotomy. *Am. J. Roentgenol.*, **143**, 37.

CRAVER, L.F. (1948). Recent advances in treatment of lymphomas, leukemias and allied disorders. *Bull. NY Acad. Med.*, **24**, 3.

CROWTHER, D., BLACKLEDGE, G. & BEST, K. (1979). The role of computed tomography of the abdomen in the diagnosis and staging of patients with lymphoma. *Clin Haematol.* **8**, 567.

JOCHELSON, M., MAUCH, P., BALIKIAN, J. *et al.* (1985). The significance of the residual mediastinal mass in treated Hodgkin's disease. *J. Clin. Oncol.*, **3**, 637.

KAPLAN, H.S., BAGSHAW, M.A. & ROSENBERG, S.A. (1964). Presentation du protocol d'essai radiotherapeutiques des lymphomes malins de L'Universite Stanford. *Nouv. Rev. Fr. Hematol.*, **4**, 95.

LISTER, T.A., CROWTHER, D., SUTCLIFFE, S.B. *et al.* (1989). Report of a committee convened to discuss the evaluation and staging of patients with Hodgkin's disease. *J. Clin. Oncol.*, **7**, 1630.

LONGCOPE, W.T. & MCKALPIN, K.R. (1920). Hodgkin's disease. *Oxford Med.*, **4**, 1.

PETERS, V.M. (1950). A study of survivals in Hodgkin's Disease treated radiologically. *Am. J. Roentgenol.*, **63**, 299.

PETERS, V.M. & MIDDLEMISS, K.C.H. (1958). A study of Hodgkin's disease treated by irradiation. *Am. J. Roentgenol.*, **79**, 114.

RADFORD, J.A., COWAN, R.A., FLANAGAN, M. *et al.* (1988). The significance of residual mediastinal abnormality on the chest radiograph following treatment for Hodgkin's disease. *J. Clin. Oncol.*, **6**, 940.

REED, D.M. (1902). On the pathological changes in Hodgkin's disease with especial reference to tuberculosis. *Johns Hopkins Rep.*, **10**, 133.

ROSENBERG, S.A. (1966). Report of the committee of the staging of Hodgkin's disease. *Cancer Res.*, **26**, 1310.

ROSENBERG, S.A. & KAPLAN, H.S. (1966). Evidence for an orderly progression in the spread of Hodgkin's disease. *Cancer Res.*, **26**, 1225.